# Pharmacists as Immunizers: The Role of Pharmacies in Promoting Immunization Campaigns and Counteracting Vaccine Hesitancy

**DOI:** 10.3390/pharmacy7040166

**Published:** 2019-12-05

**Authors:** Nicola Luigi Bragazzi

**Affiliations:** Department of Mathematics and Statistics, York University, 4700 Keele Street, Toronto, ON M3J 1P3, Canada; bragazzi@yorku.ca

**Keywords:** community pharmacies, vaccination campaign, vaccine hesitancy, evidence-based public health and vaccinology

## Abstract

Vaccines represent fundamental public health interventions aimed to counteract or, at least, partially mitigate the severe epidemiological and economic burden generated by communicable disorders, in terms of (i) outcome-related, (ii) behavior-related productivity gains, and (iii) community externalities in developed settings as well as in developing countries. Despite their importance, several parents choose not to immunize their children due to the rising phenomenon of anti-vaccination movements that divulge vaccine-related “fake news” and “post-modern, post-factual truths”. Vaccine hesitancy represents a threat that can seriously jeopardize the implementation and success of vaccination campaigns. Within this framework, from a public health perspective, community pharmacies can play a vital role in that pharmacists can: (i) act as immunizers (vaccine distributors, educators, facilitators and administrators), (ii) improve vaccine-related health literacy and vaccination coverage rates as well as (iii) remove barriers and obstacles to the access to healthcare settings offering immunization services and (iv) counteract vaccine hesitancy.

## 1. Introduction

Vaccines represent fundamental public health interventions aimed to counteract or, at least, partially mitigate the severe epidemiological and economic burden generated by communicable disorders, in terms of (i) outcome-related, (ii) behavior-related productivity gains, and (iii) community externalities in developed settings as well as in developing countries [1,2,3]. Vaccines offer both direct and indirect protection against infectious disorders (the so-called “herd immunity effect”) [4].

Despite their importance, several parents choose not to immunize their children due to the rising phenomenon of anti-vaccination movements that divulge vaccine-related “fake news” and “post-modern, post-factual truths”. Vaccine hesitancy represents a threat that can seriously jeopardize the implementation and success of vaccination campaigns [5]. Concerns and/or misinformation and misconceptions concerning immunization practices may result in a series of behaviors that delay, postpone, or even refuse vaccination. As a consequence, low, suboptimal vaccine uptake makes the achievement of an adequate vaccination coverage and the attainment of herd immunity unfeasible, which can potentially lead to pathogen re-emergence and disease outbreaks. Therefore, as stated by the “Strategic Advisory Group of Experts” (SAGE), it is of paramount importance to identify the determinants of compliance to the immunization practice: namely, (i) access to healthcare facilities, (ii) affordability of the vaccine, (iii) awareness of the importance of the vaccination, (iv) its acceptance, and (v) activation (the so-called “5A taxonomy”) [6], or in other words, complacency, convenience and confidence (the so-called “3C model”) [7].

Within this framework, from a public health perspective, community pharmacies can play a vital role in that pharmacists can: (i) act as immunizers, (ii) improve vaccine-related health literacy and vaccination coverage rates as well as (iii) remove barriers and obstacles to the access to healthcare settings offering immunization services and (iv) counteract vaccine hesitancy. 

## 2. Pharmacists as Immunizers 

Usually, vaccination campaigns are led by public health physicians, pediatricians, primary care providers, and nurses. Recently, in order to increase the vaccination coverage rate, other allied health professionals and figures including community pharmacists and pharmacist-extenders like technicians, pharmacy students and interns have been involved in immunization programs. 

Despite dating back to the seventies [8], this idea has not been implemented until recently and, due to jurisdiction, regulatory, political, and social differences, the degree and the extent to which community pharmacists or similar professional figures are fully involved in immunization programs can vary according to the country. For instance, in the USA, community pharmacists have been involved in the offer of “pharmacy-based immunization services” (PBIS) since 1984. This involvement has been further strengthened in 1995–1996, thanks to a project that has been fully implemented in 2009 for all 50 states in the USA [9]. Similar programs and initiatives have been reported and described in Portugal and France. 

Pharmacists as immunizers can play different roles: namely, (i) vaccine distributors, (ii) educators, (iii) facilitators, and (iv) administrators, as briefly overviewed in Table 1 [10,11].

A recently published systematic review of the literature [9] has been conducted to explore the feasibility, acceptability, and general effectiveness of the PBIS. This review was carried out mining several scholarly databases including PubMed/MEDLINE, EMBASE, Scopus, the Cochrane Libraries, and the “Latin American and Caribbean Health Sciences Literature Database” or LILACS. A sample of 47 studies were included, showing that PBIS are generally highly accepted both by patients and community pharmacy staff members and are effective in removing barriers and obstacles to the access of healthcare facilities and settings, offering immunization practices and related services. However, although several efforts have been made from the regulatory and training perspectives, some political and organizational gaps remain that should be properly addressed in order to further strengthen the sustainability and the role of PBIS. 

According to another recent systematic review of the literature and meta-analysis [10,11], conducted by mining several scholarly databases (including PubMed/MEDLINE, EMBASE, the Cochrane Libraries, the Cumulative Index to Nursing and Allied Health Literature or CINAHL, the International Pharmaceutical Abstracts and Google Scholar for the gray literature), pharmacists, acting as educators/facilitators or as administrators, can improve the number and success of immunization campaigns with a relative risk (RR) of 2.96 ([95% CI 1.02–8.59], k = 22 studies) and of 2.64 ([95% CI 1.81–3.85], k = 14 studies), respectively. Overall, acting either as educators/facilitators or administrators, there is a general positive effect of the role of pharmacists as immunizers on vaccine uptake and immunization coverage rate (RR 2.74 [95% CI 1.58–4.74]). 

However, several research gaps remain to be assessed. For example, several studies have investigated the effect of pharmacist involvement on influenza and/or pneumococcal vaccine uptake, whereas the effectiveness of PBIS for other vaccine products remains unknown and largely overlooked. Only one study has explored the feasibility and effectiveness of PBIS offering tetanus, diphtheria, and pertussis immunization. 

Other gaps are methodological, with most investigations being pilot studies using small sample sizes or being non-randomized. There exist in the literature only three cluster randomized and six randomized controlled clinical trials.

From an economic standpoint, a recent study conducted in Ontario (Canada) showed that, after two influenza seasons, the implementation of the PBIS resulted in savings calculated to be up to $2.3 million in direct health care costs and lost productivity [12].

Table 2 provides an overview of the available scientific evidence. 

## 3. Pharmacists and Vaccination Campaigns: Current Ongoing and Future Projects

Vaccination campaigns administered by community pharmacists are a hot topic, which is attracting considerable interest from the scientific community [13,14].

The “International Pharmacists-as-Immunizers Partnership” (IPIP), an international network of pharmacy practice researchers with a strong interest and commitment toward pharmacist-led immunizations, is a particularly important ongoing initiative. A recent two-day meeting has been held at the University of Waterloo, Waterloo (Canada), gathering prominent scientists from various countries, summarizing the current state-of-art, and identifying pitfalls and new venues in the research field of PBIS [15].

## 4. Conclusions

This Special Issue entitled “Pharmacists as Immunizers: The Role of Pharmacies in Promoting Immunization Campaigns and Counteracting Vaccine Hesitancy” welcomes both original and review papers addressing the important topic of community pharmacists as crucial actors in the promotion of vaccination campaigns. Original articles, randomized trials, and systematic reviews of the literature with meta-analysis addressing the above-mentioned science gaps are especially welcome.

## Figures and Tables

**Table 1:** The role of community pharmacists as immunizers.

Role of Pharmacist as Immunizer	Details
Distributor	Supply and distribute vaccines and immunization products
Educator	Improve vaccine-related literacy Promote the importance of vaccination and increase the awareness of the benefits of the immunization practice and vaccine uptake
Facilitator	Hosting healthcare providers (pharmacy-based clinics and similar health-related settings)
Administrator	Directly immunize subjects

**Table 2:** The available scientific evidence concerning the involvement of pharmacists as immunizers.

Impact of Pharmacist as Immunizer	Details	Reference
Feasibility, acceptability and general effectiveness of PBIS	Highly accepted by patients and community pharmacy staff members	[9]
Vaccine uptake and immunization coverage rate	RR from 2.64 [95% CI 1.81–3.85] to 2.96 [95% CI 1.02–8.59]; pooled RR 2.74 [95% CI 1.58–4.74]	[10,11]
Economic savings	$2.3 million saved in direct healthcare costs and lost productivity at the province level	[12]
